# On the Accuracy of Describing Polyelectrolyte Systems Based on Cross-Linked Networks in Terms of Linear Differential Equations

**DOI:** 10.3390/polym18050635

**Published:** 2026-03-04

**Authors:** Dina Shaltykova, Eldar Kopishev, Gaini Seitenova, Ibragim Suleimenov

**Affiliations:** 1 National Engineering Academy of the Republic of Kazakhstan, Almaty 050010, Kazakhstan; shaltykova.d@mail.ru; 2 Department of Chemistry, L.N. Gumilyov Eurasian National University, Astana 010000, Kazakhstan; seitenova_gzh@enu.kz

**Keywords:** polyelectrolyte hydrogels, linear differential equations, Poisson–Boltzmann equation, Debye length, electric double layer, non-equilibrium systems, organic electronics

## Abstract

Theoretical models of polyelectrolyte systems with cross-linked polymer networks are often simplified to linear differential equations by means of the linearized Poisson–Boltzmann approximation, whose validity is traditionally limited to cases where the electrostatic potentials are small. However, the limits of applicability of the linear theory remain debatable in many cases. Moreover, the Poisson–Boltzmann equation is, in principle, not applicable to the description of non-equilibrium systems, particularly those through which an electric current flows. In the present work, a direct comparison is carried out between the exact solution and the approximate solution (i.e., the solution obtained within the framework of the linearization procedure) of the equations describing the contact region between a cross-linked polyelectrolyte network and a low-molecular-mass salt solution. This makes it possible to determine the conditions under which the linear model is applicable, including for the analysis of promising systems in the field of organic electronics. The conclusions obtained in this work are based on basic electrostatics equations and transport equations of low-molecular-mass ions. The proposed approach also makes it possible to obtain a generalized linear differential equation that is not subject to a Boltzmann distribution approximation and is valid for polyelectrolyte systems rather far from thermodynamic equilibrium and even carrying steady electric currents.

## 1. Introduction

Researchers have always been interested in studying polyelectrolytes [1]. The primary biological information macromolecules, DNA and RNA, are also polyelectrolytes, which greatly influences this interest [2]. The Debye length [3], which describes the spatial scale over which deviations from electroneutrality may be observed, has been and continues to be one of the primary quantitative parameters in polyelectrolyte physics. All sciences that work with macroscopic objects whose behaviour is controlled by ion motion—including thermal motion—use the Debye length concept. This is especially true for plasma physics [4]. The linearization process of the Poisson–Boltzmann equation, which describes the spatial distribution of charges in situations where inhomogeneous electrostatic fields form in the system under consideration, including those caused by the thermal motion of low-molecular-weight ions, is closely related to this idea (including historically [5]). The development of electric double layers at the interface between a low-molecular-weight salt solution and a cross-linked polyelectrolyte network serves as an illustration in this regard [6].

Obtaining solutions in the most general cases is made convenient by the linearization process, but these solutions are necessarily approximations. However, this kind of explanation is frequently adequate since it gives insight into how the system in question operates. Classical electronics is a prime example in this respect, where, for example, using a rough model of a semiconductor transistor is frequently adequate [7]. However, in these situations, the issue of the model’s applicability boundaries becomes crucial.

The following factors make analogies with electronics relevant to the theoretical description of polyelectrolyte-based systems. The study of physicochemical systems based on polyelectrolyte networks is of considerable interest, from the point of view of creating and improving neuromorphic materials [8,9,10,11,12]. Such materials are becoming particularly relevant in connection with the growing use of AI in various fields of activity [9,13,14,15]. This, in turn, leads to an increase in energy consumption on a global scale [16,17,18]. Neuromorphic materials have the potential to enable the implementation of AI that requires significantly less energy, as their use [18], among other things, allows one of the main drawbacks of von Neumann architecture, which is associated with the need for continuous data exchange between the computational units themselves and the memory cells [19]. Currently, several promising neuromorphic materials are being discussed in the literature, for example, organics, metal oxides, low-dimensional materials, and perovskite materials [20,21,22]. Alongside these, there are many works, in particular [23,24], which propose neuromorphic materials based on organic electronics [25,26], including those using cross-linked polymer networks [27,28,29]. The theoretical description of such systems requires solving problems like those known from the theory of electrolytes [30] and polyelectrolytes [31,32]. Determination of electrical characteristics of any system using polyelectrolyte networks requires establishing the distribution of electric fields (or potentials) in such a system, which raises the question of the adequacy of using the linearized form of the Poisson–Boltzmann equation.

One of the main questions that arises when using the linearization procedure is, of course, the question of the accuracy of such an approximation; this point should be emphasized once again. Let us recall the idea of deriving the linearized form of the Poisson–Boltzmann equation, considering for clarity the case of a symmetric 1:1 electrolyte.

The electrostatic equations used, which in the SI system can be written as

(1) $$\varepsilon_{0}\varepsilon \nabla \vec{E}=\rho$$

(2) $$\vec{E}=-\nabla \varphi$$

where ρ is the distribution of electric charges, φ is the electric potential, $\varepsilon_{0}$ and ε are the permittivities of vacuum and of the medium, respectively, and $\vec{E}$ is the electric field strength vector.

(3) $$\nabla =\vec{i}\frac{\partial }{\partial x}+\vec{j}\frac{\partial }{\partial y}+\vec{k}\frac{\partial }{\partial z}$$

It is assumed that the charge distribution in space obeys the Boltzmann distribution; thus, for a 1:1 electrolyte, the following expression applies.

(4) $$\rho =e\left(n_{+}-n_{-}\right)=e\left(n_{+0}\exp-\frac{e\varphi }{kT}-n_{-0}\exp\frac{e\varphi }{kT}\right)$$

where $n_{\pm }$ is the concentration of ions of the corresponding sign, $n_{\pm 0}$ is the concentration at the point from which the potential is measured, e is the elementary charge, k is the Boltzmann constant, and T is the absolute temperature.

Expanding the exponential terms in a Taylor series and retaining only the linear terms,

(5) $$\exp\pm \frac{e\varphi }{kT}\approx 1\pm \frac{e\varphi }{kT}$$

Thus, we obtain

(6) $$\nabla^{2}\varphi =\frac{1}{\lambda^{2}}\varphi$$

where $\lambda =\sqrt{\frac{\varepsilon_{0}\varepsilon kT}{2n_{0}e^{2}}}$ is the Debye length.

The applicability of expansion (5) is governed by an obvious criterion.

(7) $$\frac{e\varphi }{kT}\ll 1$$

It is also evident that its implementation imposes very strict limitations on the nature of the system, allowing for a transition to a description in terms of linear differential equations.

The use of the linearized Poisson–Boltzmann equation in many cases yields very useful results [33,34,35]; however, it is, in principle, not applicable, for example, to polyelectrolyte-based systems through which an electric current flows. At the same time, it is precisely such systems that are of interest from the standpoint of organic electronics, the physical chemistry of neuromorphic and sociomorphic materials, etc. [23,36,37]. Therefore, it is of interest to obtain a generalization of Equation (6) for this case. It is evident that in this situation it is no longer possible to employ the Boltzmann distribution to describe the dependence of charged particle concentrations on the electric field profile, which constitutes the basis of the classical Debye–Hückel treatment, where a spherically symmetric cloud screening an isolated electrostatic charge is considered.

This paper shows that it is possible to obtain a more general form of Equation (6), which is valid, among other things, for the case when the system under consideration is far from thermodynamic equilibrium, for example, for the case when an electric current flows through it. As follows from the above, the solution of such problems is relevant, among other things, for organic electronics and neuromorphic systems based on it.

In this context, the question of the accuracy of the description within the framework of the linearization procedure naturally becomes particularly relevant. This work also proves that the generalised form of Equation (6) describes the processes occurring in systems based on polyelectrolyte hydrogels with sufficient accuracy for practical use (including for solving problems in the field of organic electronics).

A direct comparison was made between the exact solution of several problems arising in the theoretical description of systems based on polyelectrolyte hydrogels and the solution obtained using the linearized equation.

## 2. Results

### 2.1. Derivation of the Basic Equation

An equation that solves the same problem as the linearized Poisson–Boltzmann equation, but in a more general form, can be derived directly from the equations of electrostatics and the equations of motion of low-molecular-weight ions.

The equations of motion for low-molecular-weight ions may be written in the following form:

(8) $$\frac{\partial n_{i}}{\partial t}+\nabla {\vec{J}}_{i}=Q_{i}$$

(9) $${\vec{J}}_{i}=-D_{i}\nabla n_{i}+z_{i}en_{i}b_{i}\vec{E}$$

where $n_{i}$ is the concentration of ions of the i-th type, ${\vec{J}}_{i}$ is the flux of such ions, $Q_{i}$ represents the spatial distribution of their sources, $z_{i}$ is the charge number, e is the elementary charge, and $D_{i}$ and $b_{i}$ are the diffusion coefficient and the mobility, respectively. We make use of the Einstein relation.

(10) $$kTD_{i}=b_{i}$$

Then, from (8) and (9), it follows that

(11) $$-\nabla^{2}n_{i}+z_{i}e\frac{1}{kT}\nabla \left(n_{i}\vec{E}\right)=0$$

The charge distribution can be expressed in terms of the concentrations of mobile charge carriers as

(12) $$\rho =e\sum_{i}z_{i}n_{i}$$

Then, multiplying Equation (11) by $z_{i}e$ and summing over all species, we obtain

(13) $$-\nabla^{2}\rho +\frac{1}{\;kT}\nabla \left(\sum_{i}z_{i}^{2}e^{2}n_{i}\vec{E}\right)=0$$

or

(14) $$-\nabla^{2}\rho +\frac{\sum_{i}z_{i}^{2}e^{2}n_{i}}{kT\varepsilon_{0}\varepsilon }\nabla \vec{E}+\frac{1}{kT}\left(\sum_{i}z_{i}^{2}e^{2}\nabla n_{i}\right)\vec{E}=0$$

The model corresponding to the system of equations written above is, strictly speaking, of limited applicability (in particular, it does not consider phenomena such as concentration polarization [28] or strong nonlinear coupling [28]). Methodologically, however, its limits of applicability fully coincide with the limits of applicability of the model based on the use of the Poisson–Boltzmann equation, which, as noted above, has demonstrated good practical performance [28].

This can be shown as follows. Equations (1) and (2) follow from the fundamental equations of electrostatics. Consequently, the limits of applicability of the model employed are effectively determined by the limits of applicability of the ion transport equation of the form (11) and the Einstein relation (10).

For the case in which the system parameters depend on only one spatial coordinate, Equation (11) may be written in the form

(15) $$\frac{d}{dx}\left[-\frac{d}{dx}n_{i}+\frac{z_{i}e}{kT}n_{i}E\right]=0$$

or

(16) $$-\frac{d}{dx}n_{i}+\frac{z_{i}e}{kT}n_{i}E=J$$

The integration constant J, in the case where the system parameters depend on only one coordinate, has the meaning of the electric current flowing through the system (up to a constant factor). If no electric current flows, this constant is equal to zero.

Equation (16) admits an analytical solution that coincides with the Boltzmann distribution for the case under consideration:

(17) $$n_{i}\left(x\right)=n_{i00}\left[-\frac{z_{i}e}{kT}\varphi \left(x\right)\right]$$

where $\varphi \left(x\right)$ is the potential profile related to the electric field profile by relation (2), and $n_{i00}$ is the concentration of mobile charges of the given type at the point where $\varphi \left(x\right)=0$.

Therefore, from a methodological standpoint, the limits of applicability of the approach employed in the present work and the limits of applicability of models based on the use of the Poisson–Boltzmann equation indeed coincide.

The standard linearization procedure, which is used not only in the physical chemistry of electrolytes but also in plasma physics [38], assumes that all parameters characterising the behaviour of the system can be specified in the form

(18) $$X_{j}=X_{0j}+\delta X_{j};\delta X_{j}\ll X_{0j}$$

The linearized system of equations includes only terms that are linear in the variations $\delta X_{j}$; that is, terms containing products $\delta X_{j}\delta X_{k}$ (or other terms quadratic in $\delta X_{j}$, $\delta X_{k}$), are neglected.

With reference to the model under consideration, Equation (18) takes the form

(19) $$\vec{E}={\vec{E}}_{0}+\delta \vec{E}$$

(20) $$n_{j}=n_{j0}+\delta n_{j}$$

where $\delta n_{j}\ll n_{j0}$, $\left\lceil \delta \vec{E}\right\rceil \ll \left\lceil {\vec{E}}_{0}\right\rceil$; $n_{j0}=const$; ${\vec{E}}_{0}=const$.

These relations imply that the “unperturbed” medium is homogeneous (in particular, the electric field in it is homogeneous), and the true solutions may be regarded as relatively small deviations from constant values.

Substituting these expressions into (13) and neglecting quadratic terms within the framework of the linearization procedure, we obtain

(21) $$-\nabla^{2}\delta \rho +\frac{1}{T}\sum_{i}z_{i}^{2}e^{2}n_{i0}\nabla \delta \vec{E}+\frac{1}{T}\left({\vec{E}}_{0}\nabla \right)\left(\sum_{i}z_{i}^{2}e^{2}\delta n_{i}\right)=0$$

or

(22) $$-\nabla^{2}\delta \rho +\frac{\sum_{i}z_{i}^{2}e^{2}n_{i0}}{kT\varepsilon_{0}\varepsilon }\delta \rho +\frac{1}{T}\left({\vec{E}}_{0}\nabla \right)\left(\sum_{i}z_{i}^{2}e^{2}\delta n_{i}\right)=0$$

or the case in which the unperturbed medium is electrically neutral, i.e., δρ=ρ, the variation symbol δ in Equation (21) may be omitted. Assuming that the unperturbed electric field is also zero, ${\vec{E}}_{0}=0$, we obtain

(23) $$\nabla^{2}\rho =\frac{\sum_{i}z_{i}^{2}e^{2}n_{i0}}{kT\varepsilon_{0}\varepsilon }\rho$$

The coefficient in Equation (23) is precisely equal to the inverse square of the Debye length

(24) $$\lambda^{2}=\frac{kT\varepsilon_{0}\varepsilon }{\sum_{i}z_{i}^{2}e^{2}n_{i0}}$$

Consequently,

(25) $$\nabla^{2}\rho =\frac{1}{\lambda^{2}}\rho$$

It should be emphasized that Equation (25) is valid not only in the case ${\vec{E}}_{0}=0$. This can conveniently be demonstrated by considering the case of a 1:1 salt solution, when the system contains only one type of singly charged cations and one type of singly charged anions. In this case, Equation (22) takes the form

(26) $$\nabla^{2}\rho =\frac{1}{\lambda^{2}}\rho +\frac{1}{\Lambda }\left({\vec{e}}_{0}\nabla \right)\rho$$

where ${\vec{e}}_{0}$ is a dimensionless unit vector whose direction coincides with that of the electric field vector, and $\Lambda^{-1}=2\frac{eE_{0}}{kT}$, _T_.e. Λ is the distance over which the potential drop equals one-half of kT.

Consequently, if the electric field strength generated, for example, by external current sources, is sufficiently small, the second term on the right-hand side of (26) may also be neglected, which leads to Equation (25). It should also be noted that there exist practically important problems for which one may assume ${\vec{E}}_{0}=0$. One example is a desalination system that utilizes the spontaneous generation of an electromotive force when a low-molecular-mass salt solution flows through a cross-linked polyelectrolyte network [28]. Another example is the problem of the formation of inhomogeneous electric fields under inhomogeneous heating. Such a problem arises when gels are used as carriers of aromatic substances in aromatherapy applications [39].

In fact, Equation (25) is fourth order, which can be verified by moving from charge distribution to potential distribution.

(27) $$\nabla^{2}\left(\nabla^{2}\varphi \right)=\frac{1}{\lambda^{2}}\nabla^{2}\varphi$$

If the term proportional to $E_{0}$ cannot be neglected, Equation (22) should be used, which, as can be seen, is also a fourth-order equation (if the charge density profile is expressed through the electrostatic potential profile).

The difference between Equations (22) and (27) and the classical form of the Poisson–Boltzmann Equation (6) is that Equations (22) and (27) also describe systems through which steady electric currents flow. This makes it useful for the theoretical description of organic electronic devices that use, for example, polyelectrolyte hydrogels.

The advantages of this form of theoretical description of systems based on polyelectrolyte networks are obvious: many problems can be solved analytically. However, an important question arises about the accuracy of such a description. This question can be resolved, among other things, by directly comparing the exact solutions of the original system of nonlinear equations with the solutions of the linearized system.

Let us consider a specific example of a system based on a polyelectrolyte hydrogel that allows for such a comparison. We consider a planar sample of a polyelectrolyte hydrogel (a sodium polyacrylate-based gel or its analogues) placed in a solution of a 1:1 electrolyte whose cations are identical to those produced by dissociation of the functional groups of the polymer network (for example, sodium chloride).

### 2.2. Comparison of the Exact and Approximate Solutions

In this case, the distributions of ion concentrations in the solution above the gel obey the following system of equations:

(28) $$-\frac{dn^{+}}{dx}+\frac{e}{kT}En^{+}=\frac{e}{kT}E_{0}n_{0}^{+}$$

(29) $$-\frac{dn^{-}}{dx}-\frac{e}{kT}En^{-}=-\frac{e}{kT}E_{0}n_{0}^{-}$$

(30) $$\varepsilon_{0}\varepsilon \frac{d}{dx}E=e\left(n^{+}-n^{-}\right)$$

where $n^{+}$ and $n^{-}$ are the concentrations of positive and negative ions, respectively; the quantities $\frac{e}{kT}E_{0}n_{0}^{+}$ may be regarded as constants of integration of Equation (11).

The quantity $E_{0}$ may be interpreted as the strength of a uniform electrical field in the above gel solution. If no electric current flows through the system, this quantity is equal to zero.

The distributions of ion concentrations within the bulk of the gel obey the same system of equations; however, Equation (30) is replaced by the following equation, which accounts for the non-zero charge of the polymer network $N_{0}$

(31) $$\varepsilon_{0}\varepsilon \frac{d}{dx}E=en^{+}-n^{-}-N_{0}$$

The principal characteristics of the system under consideration can be obtained without solving the nonlinear differential equations [40]. By adding Equations (28) and (29), we obtain

(32) $$-\frac{d}{dx}\left(n^{+}+n^{-}\right)+\frac{e}{kT}E\left(n^{+}-n^{-}\right)=0$$

The last term in Formula (32) can be expressed in terms of the electric field strength using relations (30) or (31) for the solution and gel, respectively. For the solution, we obtain

(33) $$-\frac{d}{dx}\left(n^{+}+n^{-}\right)+\frac{\varepsilon_{0}\varepsilon }{kT}E\frac{d}{dx}E=0$$

Regarding the gel volume, we obtain

(34) $$-\frac{d}{dx}\left(n^{+}+n^{-}\right)+\frac{\varepsilon_{0}\varepsilon }{kT}E\frac{d}{dx}E+\frac{e}{kT}EN_{0}=0$$

Equations (33) and (34) integrate directly. We obtain

(35) $$n^{+}+n^{-}-\frac{\varepsilon_{0}\varepsilon }{2kT}E^{2}=C_{1}$$

(36) $$\left(n^{+}+n^{-}\right)-\frac{\varepsilon_{0}\varepsilon }{2kT}E^{2}+\frac{e}{kT}N_{0}\varphi =C_{2}$$

From Equations (27) and (28), it follows that

(37) $$n^{+}(0)+n^{-}(0)-\frac{\varepsilon_{0}\varepsilon }{2kT}E_{0}^{2}=n_{s}^{+}+n_{s}^{-}$$

(38) $$n^{+}\left(0\right)+n^{-}\left(0\right)-\frac{\varepsilon_{0}\varepsilon }{2kT}E_{0}^{2}+\frac{e}{kT}N_{0}∆\varphi =n_{g}^{+}+n_{g}^{-}$$

where $n^{+}\left(0\right)$ and $n^{+}\left(0\right)$ are concentrations of positive and negative ions at the gel boundary, respectively, the indices s and g denote the ion concentrations in the bulk of the solution and the gel, respectively, and $E_{0}$ is the electric field at the gel–solution interface.

From the condition of continuity of the electric field at the solution–gel interface, together with Equations (37) and (38), the following expression for the potential difference between the solution and the bulk of the gel, ∆φ, is obtained.

(39) $$\frac{e}{kT}N_{0}∆\varphi =n_{g}^{+}+n_{g}^{-}-n_{s}^{+}-n_{s}^{-}$$

We rewrite expression (39) in a form that allows for subsequent quantitative comparison with the solution of the same problem in the linear approximation. We consider that the medium is electrically neutral both in the solution and within the gel, i.e.,

(40) $$n_{s}^{+}=n_{s}^{-}=c_{s}$$

(41) $$n_{g}^{-}=c_{g};n_{g}^{+}=N_{0}+c_{g}$$

where $c_{s}$ and $c_{g}$ are the concentrations of the low-molecular-weight salt in the solution and in the gel, respectively.

Then

(42) $$\frac{e}{kT}∆\varphi =1-2\frac{c_{s}-c_{g}}{N_{0}}$$

The quantities $c_{s}$ and $c_{g}$ are not independent; they are related by the Donnan equilibrium condition [34].

(43) $$c_{g}\left(c_{g}+N_{0}\right)=c_{s}^{2}$$

from which

(44) $$c_{g}=\frac{1}{2}\sqrt{N_{0}^{2}+4c_{s}^{2}}-\frac{1}{2}N_{0}$$

Therefore, the reduced dimensionless potential difference $\frac{e}{kT}∆\varphi$ may be regarded as a function of a single parameter—the reduced salt concentration in the solution above the gel, $\xi =\frac{c_{s}}{N_{0}}$

(45) $$\frac{e}{kT}∆\varphi =\frac{\sqrt{N_{0}^{2}+4c_{s}^{2}}-2c_{s}}{N_{0}}=\sqrt{1+4\xi^{2}}-2\xi$$

We now solve the same problem within the framework of the linear approximation using Equation (27). Since all functions describing the system depend on only one spatial variable, this equation can be rewritten in the form

(46) $$\frac{d^{4}}{dx^{4}}\varphi =\frac{1}{\lambda^{2}}\frac{d^{2}}{dx^{2}}\varphi$$

or

(47) $$\frac{d^{3}}{dx^{3}}E=\frac{1}{\lambda^{2}}\frac{d}{dx}E$$

Integrating Equation (47) and considering that the electric field becomes zero away from the boundary, we obtain the elementary solutions

(48) $$E\left(x\right)=E_{0}\exp\pm \frac{x}{\lambda }$$

Since the electric field is zero in the bulk of the gel and in the bulk of the solution, the signs of the solutions should be chosen as follows:

(49) $$E_{g}\left(x\right)=-E_{0}\exp\frac{x}{\lambda_{g}}$$

(50) $$E_{s}\left(x\right)=-E_{0}\exp-\frac{x}{\lambda_{s}}$$

The constant $E_{0}$ in expressions (49) and (50) is the same, since the electric field is continuous at the gel–solution interface, whereas the Debye lengths in the solution $\lambda_{s}$ and in the gel $\lambda_{g}$ differ because the concentrations of mobile ions are not the same.

(51) $$\lambda_{s}=\sqrt{\frac{\varepsilon_{0}\varepsilon kT}{2c_{s}e^{2}}}\;\;\;\;;\;\lambda_{g}=\sqrt{\frac{\varepsilon_{0}\varepsilon kT}{\left(2c_{g}+N_{0}\right)e^{2}}}$$

**Figure 1:**
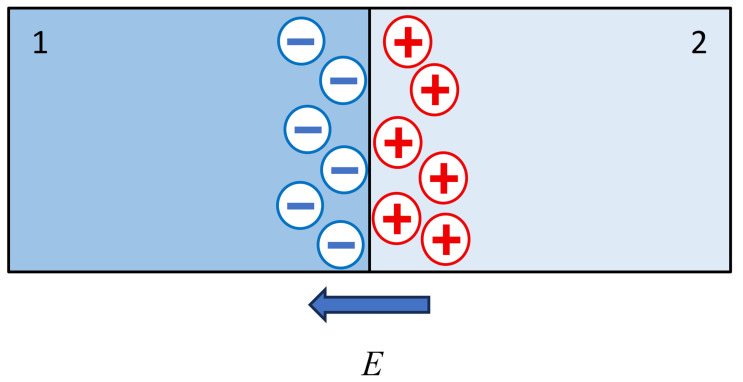
Mechanism of formation of the electric double layer at the polyelectrolyte gel–solution interface: justification of the sign choice in Equations (49) and (50).

The minus sign in Equations (49) and (50) was chosen for the following reasons. The electric field strength in systems of the type under consideration is different from zero only near the interface between the media, i.e., in the region where a double electric layer is formed (Figure 1). The formation of this layer is determined by [40] the thermal motion of counterions (for example, sodium ions, if we consider a sodium polyacrylate-based gel placed in a sodium chloride solution). Thermal motion leads to the formation of an uncompensated positive charge in the solution near the interface. This charge is held by the attraction of negative charges of functional groups, also concentrated near the surface. The electric field vector is directed from positive to negative, so with the choice of coordinate system shown in Figure 1, the field strength is negative.

This diagram illustrates the mechanism of electric double-layer formation associated with the thermal motion of low-molecular-mass ions. Specifically, under its influence, positive ions (for illustration, a sodium polysalt-based network is considered) leave the surface layer of the network (an uncompensated positive charge is formed in region 2, Figure 1). In region 1 of Figure 1, an uncompensated negative charge correspondingly remains, which prevents further migration of positive ions into the solution.

Since the electric field is zero both in the gel and in the solution, the signs of the solutions should be chosen as follows:

Combining Equations (49) and (50), we obtain

(52) $$E\left(x\right)=\left\{\begin{array}{c} -E_{0}\exp\frac{x}{\lambda_{g}},x<0\\ -E_{0}\exp-\frac{x}{\lambda_{s}},x\geq 0 \end{array}\right.$$

We choose the potential to be measured from a point located in the bulk of the gel. The potential distribution in the system under consideration is then given by the expression

(53) $$\varphi \left(x\right)=-\int_{-\infty }^{x}E\left(x\right)dx$$

In particular, the following condition must be satisfied:

(54) $$\frac{e}{kT}{∆}_{0}\varphi =-\frac{e}{kT}\int_{-\infty }^{\infty }E\left(x\right)dx=-\ln\frac{c_{s}}{c_{g}}$$

where ${∆}_{0}\varphi$ is the potential difference between the solution and the gel.

**Figure 2:**
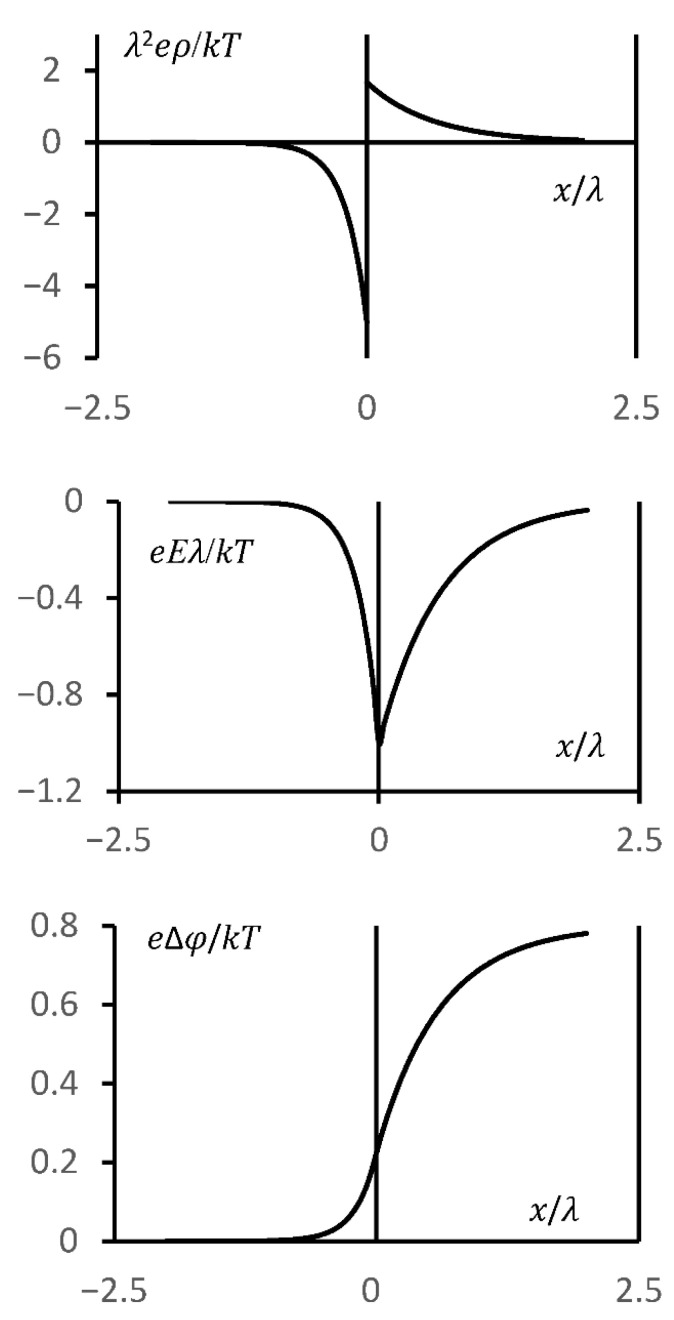
The dependences of the reduced charge $\lambda^{2}\frac{e\rho }{kT}$, the reduced electric field strength $\lambda \frac{eE}{kT}$, and the reduced potential $\frac{e∆\varphi }{kT}$ on the coordinate x/λ in the vicinity of the solution–gel interface, calculated within the linear approximation.

Based on relations (52) and (53), Figure 2 was constructed; it also illustrates the mechanism of electric double-layer formation at the interface between the polyelectrolyte gel and the solution (it should be noted that the use of a sharp interface between the cross-linked network and the solution is an idealization; this issue is discussed in more detail in Section 3). In constructing this figure, Equation (1) was also used, which allows the charge density distribution to be determined from the known field distribution in the system. One can see that the figure fully agrees with the qualitative interpretation of the double-layer formation mechanism (Figure 1). Specifically, the function $E\left(x\right)$, shown in the figure, is continuous; however, its derivative exhibits a discontinuity, which corresponds to the formation of an electric double layer. Note also that the “thickness” of the charged layers outside and inside the hydrogel is different, reflecting the difference between $\lambda_{s}$ and $\lambda_{g}$. Based on this qualitative picture, let us derive an expression for the reduced potential difference $\frac{e}{kT}∆\varphi$.

Carrying out the integration in Formula (54), we obtain

(55) $$\frac{e}{kT}\left(\lambda_{s}+\lambda_{g}\right)E_{0}=-\frac{e}{kT}\int_{-\infty }^{\infty }E\left(x\right)dx=-\ln\frac{c_{s}}{c_{g}}$$

In this case, the potential difference between the gel–solution interface and the bulk of the gel is given by

(56) $$∆\varphi =\lambda_{s}E_{0}$$

from which the reduced potential difference is given by

(57) $$\frac{e}{kT}∆\varphi =\frac{\lambda_{s}}{\lambda_{s}+\lambda_{g}}\ln\frac{c_{s}}{c_{g}}$$

Or, using the expression for the Debye length given in (51),

(58) $$\frac{e}{kT}∆\varphi =-\frac{\sqrt{2c_{g}+N_{0}}}{\sqrt{2c_{g}+N_{0}}+\sqrt{2c_{s}}}\ln\frac{c_{g}}{c_{s}}$$

Equation (58) yields the value of the same quantity as Equation (45), but within the linear approximation. For the convenience of comparison, we express the right-hand side of relation (58) in terms of the reduced concentration ξ. We obtain

(59) $$\frac{e∆\varphi }{kT}=-\frac{\sqrt[{4}]{N_{0}^{2}+4c_{s}^{2}}}{\sqrt[{4}]{N_{0}^{2}+4c_{s}^{2}}+\sqrt{2c_{s}}}\ln\frac{\sqrt{N_{0}^{2}+4c_{s}^{2}}-N_{0}}{2c_{s}}=-\frac{\sqrt[{4}]{1+4\xi^{2}}}{\sqrt[{4}]{1+4\xi^{2}}+\sqrt{2\xi }}\ln\frac{\sqrt{1+4\xi^{2}}-1}{2\xi }$$

**Figure 3:**
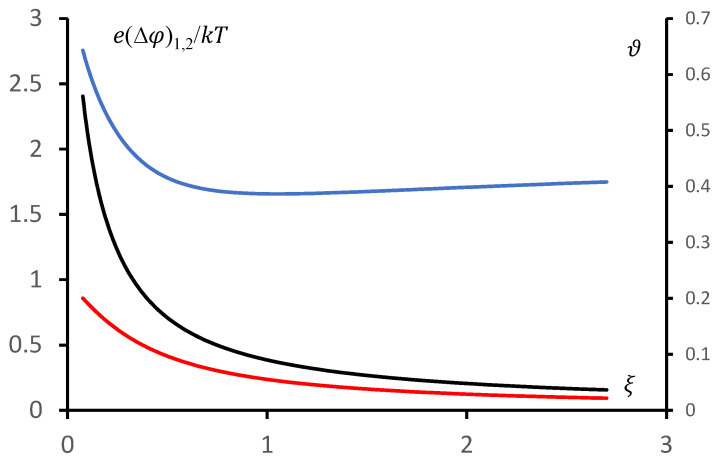
A comparison of the dependence of the reduced potential difference between the hydrogel bulk and the hydrogel surface, obtained from the exact solution $\frac{e{\left(∆\varphi \right)}_{1}}{kT}$ (curve 1, red, left axis) and from the approximate solution $\frac{e{\left(∆\varphi \right)}_{2}}{kT}$ (curve 2, black, left axis) of the original system of equations, as well as the relative deviation ϑ (curve 3, blue, right axis) on the reduced salt concentration in the solution above the gel ξ, is shown.

A comparison of the dependence of the reduced potential difference between the hydrogel bulk and the hydrogel surface, obtained from the exact solution $\frac{e{\left(∆\varphi \right)}_{1}}{kT}$ and from the approximate solution $\frac{e{\left(∆\varphi \right)}_{2}}{kT}$ of the original system of equations, on the reduced salt concentration ξ is shown in Figure 3. The corresponding relative deviation $\vartheta =\frac{{\left(∆\varphi \right)}_{1}-{\left(∆\varphi \right)}_{2}}{{\left(∆\varphi \right)}_{1}}$.

At sufficiently high salt concentrations (ξ>1), the relative deviation of the exact solution from the approximate solution remains approximately constant and is about 40%. The deviations begin to increase significantly at low values of ξ, which corresponds to sufficiently large potential differences between the gel volume and its boundary. Qualitatively, this corresponds to the fulfilment of condition (7).

The deviation from the exact solution demonstrated in Figure 3 appears, at first glance, to be significant. However, if one proceeds from the analogy with classical electronics, such a deviation may be regarded as acceptable for solving a sufficiently broad class of problems. This is discussed in more detail in Section 3.

The comparison presented above referred only to specific parameters that describe the system of the type under consideration. An exact solution for the electric field profile in such a system has not yet been obtained. Continuing the logic on which this work is based, we will compare the electric field profile obtained from the exact solution of the system of nonlinear equations with the approximate solution obtained within the framework of the linear model.

Subtracting Equations (28) and (29) from each other, we obtain

(60) $$-\frac{d}{dx}\left(n^{+}-n^{-}\right)+\frac{d}{dx}\left(n^{+}+n^{-}\right)\frac{e}{kT}E=0$$

Using relations (35) and (40), expression (60) can be rewritten in the following form:

(61) $$-\frac{\varepsilon_{0}\varepsilon }{e}\frac{d^{2}}{dx^{2}}E+\frac{e}{kT}E\left(2c_{s}+\frac{1}{2}\frac{\varepsilon_{0}\varepsilon }{kT}E^{2}\right)=0$$

The only controlling parameter in this formula is also the Debye length (which, for the solution above the gel, is given by $\lambda_{s}^{2}=\frac{\varepsilon_{0}\varepsilon kT}{2c_{s}e^{2}}$). This can be seen by rewriting expression (61) in the following form:

(62) $$-\frac{d^{2}}{dx^{2}}E+\frac{2c_{s}e^{2}}{kT\varepsilon_{0}\varepsilon }E\left(1+\frac{1}{2}\frac{{kT\varepsilon }_{0}\varepsilon }{2e^{2}c_{s}}{\left(\frac{eE}{kT}\right)}^{2}\right)=0$$

Consequently, expression (61) can be transformed into a form that contains only dimensionless quantities.

(63) $$-\frac{d^{2}}{dX^{2}}f+f\left(1+\frac{1}{2}f^{2}\right)=0$$

where $f=\lambda_{s}\frac{eE}{kT}$ is the dimensionless (reduced) electric field, $X=\frac{x}{\lambda_{s}}$ is the dimensionless coordinate.

Multiplying Equation (63) by $\frac{df}{dX}$ and integrating, we obtain

(64) $${\left(\frac{df}{dX}\right)}^{2}=f^{2}\left(1+\frac{1}{4}f^{2}\right)+C$$

The integration constant C may be set equal to zero due to the fulfilment of the condition

(65) $${\left.\frac{df}{dX}\right|}_{f\to 0}=0$$

Consequently,

(66) $$\int \frac{df}{f\sqrt{4+f^{2}}}=-\frac{1}{2}X+\frac{1}{2}X_{0}$$

The minus sign is chosen because the function f must decrease with increasing distance from the interface between the media. This integral is a particular case of the following tabulated integral (verification of the adequacy of the tabulated data is provided in the Supplementary Materials).

(67) $$\int \frac{dx}{x\sqrt{a^{2}+x^{2}}}=\frac{1}{a}\ln\frac{a\sqrt{x^{2}+a^{2}}-a^{2}}{x}$$

We obtain

(68) $$\mathrm{Ln}\frac{2\sqrt{f^{2}+4}-4}{f}=-X+X_{0}$$

from which

(69) $$f=\frac{8A\exp\left(-X\right)}{4-A^{2}\exp\left(-2X\right)}$$

The coefficient A is determined from the boundary condition, namely the value of the reduced potential at the interface between the media, f(0). We obtain

(70) $$A=\frac{2\sqrt{{f(0)}^{2}+4}-4}{f(0)}$$

**Figure 4:**
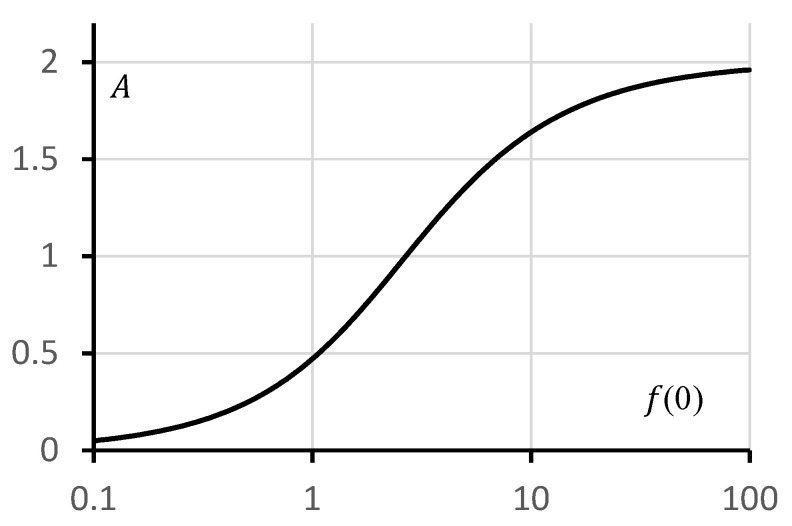
Dependence of the coefficient A on the boundary value f(0).

The dependence of the coefficient A on the boundary condition f(0) is shown in Figure 4 on a semi-logarithmic scale.

**Figure 5:**
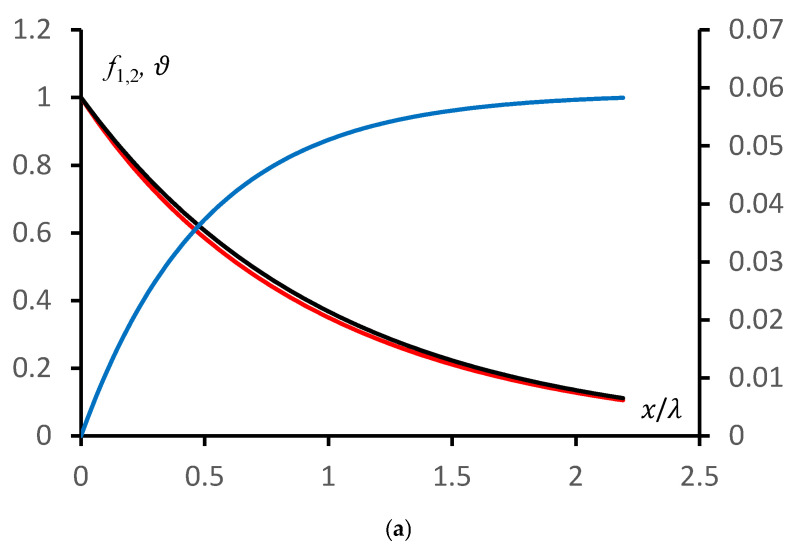

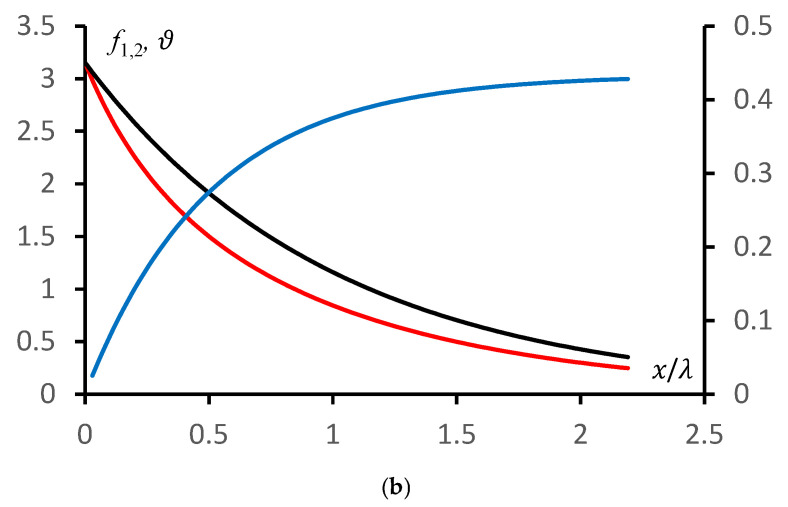
Comparison of the exact (red) and approximate (black) solutions for the reduced potential distribution near the hydrogel boundary, and the dependence of the relative deviation ϑ on the reduced coordinate; f=1.00 (**a**) f=3.15 (**b**).

The resulting curve exhibits an inflection point in the vicinity of $f\left(0\right)\approx 1$. The dependences of the exact solution $f_{1}$ and the approximate solution $f_{2}$ for the reduced potential on the reduced coordinate are shown in Figure 5. The same figure also presents the dependence of the relative deviation $\vartheta =\frac{f_{2}-f_{1}}{f_{1}}$ on the reduced coordinate. Figure 5a corresponds to a value of $f\left(0\right)$ lying below the inflection point in Figure 4, whereas Figure 5b corresponds to the opposite case. It can be observed that for relatively small values of $f\left(0\right)$, the approximate and exact solutions practically coincide. Noticeable deviations appear only at comparatively large values of $f\left(0\right)$.

## 3. Discussion

Thus, it becomes possible to estimate the accuracy of models based on the use of linearized equations describing systems based on cross-linked polyelectrolyte networks through which an electric current flows.

Naturally, the comparison between the exact and the approximate solutions presented above refers to a particular problem in which a sample of a cross-linked network placed in a 1:1 low-molecular-mass salt solution is considered. However, proceeding from the analogy with classical electronics, such a problem may be used to analyze a wide range of systems related to organic electronics, neuromorphic materials, etc. Indeed, contact phenomena at the cross-linked network–solution interface (or in the analogous problem corresponding to the contact region of two oppositely charged networks) are largely analogous to the contact phenomena occurring in the p–n or n–p junction region, which, in turn, constitute the basis of operation not only of classical transistors but also of the most complex integrated circuits: the equivalent circuits of integrated circuits reduce to a set of transistors interconnected by contacts. At the same time, the transistors themselves effectively represent separate regions of a semiconductor crystal processed, for example, by photolithography. Consequently, there are strong grounds to assume that the above-mentioned contact region represents one of the key objects of organic electronics, which is also supported by the opinion of the authors [28].

The analogy with classical semiconductor electronics also makes it possible to assert that, in many cases, the accuracy of the approximate description obtained within the framework of the linearization procedure is entirely acceptable. Indeed, a rigorous model of semiconductor transistor operation should, strictly speaking, be constructed taking into account quantum mechanical effects, since the “holes” responsible for the formation of n–p–n or p–n–p structures are correctly interpreted as quasiparticles, whose behavior is determined by the energy band structure of a doped semiconductor crystal [41]. In practice, however, highly simplified models of semiconductor transistors are used.

One of the most well-known examples is the model of the classical semiconductor transistor presented in the book [7]. It reduces the description of transistor operation to two statements

- No current flows through the base;
- The voltage drop between the base and the emitter is approximately 0.6 V.

In practice, the latter value may vary within a wide range (especially in percentage terms); however, such variations are not essential for circuits designed even based on this highly simplified model.

Moreover, from the standpoint of classical electronics, complex models represent an excessive level of accuracy, since technological variability in the fabrication even of individual transistors is quite significant. Therefore, the use of a high-precision model is not necessary from the viewpoint of practical application. This constitutes one of the main arguments in favor of using maximally simple and transparent models in classical electronics. There are strong grounds to assume that a similar situation arises in organic electronics.

Similar considerations apply to the correctness of using a model with sharp hydrogel–solution interfaces. Such an interface is diffuse in nature. This is especially true when the interfaces between phases are poorly defined, as happens, for instance, when cross-linked networks interact with polyvalent metal ions [42,43].

Furthermore, there are strong grounds to assume that the linearized model will yield more accurate results when the system characteristics (in particular, the network charge density) vary sufficiently smoothly. This follows from the results presented in Figure 2. They show that the most pronounced jumps in electrical characteristics occur when the network charge density changes abruptly.

Another conclusion that follows directly from the comparison presented above can be formulated as follows. Solutions of the exact equations describing boundary or contact phenomena in media of the type under consideration can, with acceptable accuracy, be approximated by exponential dependences, even under conditions where the variation in the governing parameters is sufficiently sharp. In other words, even in cases where the above-mentioned criterion of small potential variation is poorly satisfied, approximation by exponential functions yields acceptable accuracy. In this case, however, the values of the parameter (the Debye length) that defines the exponential solutions may differ substantially from those obtained from the solution of the linearized equation.

This can be illustrated by a specific example. The curves presented in Figure 5, corresponding to the exact solution of Equation (64), are approximated by an exponential function with high accuracy; however, the value of the governing parameter, i.e., the Debye length, must be adjusted.

In the long term, this conclusion makes it possible to develop the following approach to solving problems related to determining the potential distribution in a system containing different phases, for example, a polyelectrolyte network phase and a solution phase. The basis of this method is the transition from differential equations to their variational form. For the equation describing the system considered above (56), the variational form can be written as follows.

(71) $$J=\int {\left(\frac{df}{dX}\right)}^{2}+f^{2}\left(1+\frac{1}{4}f^{2}\right)dX$$

This can be demonstrated by directly applying classical methods of the calculus of variations to the equation written above. Specifically, the function appearing under the integral sign in (63) can be represented in the following form:

(72) $$F\left(f,g\right)=g^{2}+f^{2}\left(1+\frac{1}{4}f^{2}\right);g=\frac{df}{dX}$$

In Euler’s equation

(73) $$\frac{d}{dX}\left(\frac{dF}{dg}\right)-\frac{dF}{dg}=0$$

functional (63) exactly coincides with Equation (56).

Furthermore, to solve the variational problem, it is not necessary to employ methods that involve finding extremals as solutions of differential equations. One can use a direct approximation of the sought solutions with undetermined parameters. This approach was implemented, among others, in the work [38] cited above. In this case, it becomes possible to determine the values of the desired parameters directly by passing the stage of solving linear differential equations, since finding the solution effectively reduces solving algebraic equations that allow one to find the extremum of a functional of the type described above.

Indeed, if the desired solution depends on a set of control parameters $a_{i}$ and its form is known, then integration can be performed

(74) $$J=\int f\left(X,a_{1},\ldots ,a_{n}\right)dX,$$

This will give us the function $J=J\left(a_{1},\ldots ,a_{n}\right)$. By finding the extremum of this function, we obtain a system of algebraic equations, which in general has the form

(75) $$\frac{d}{da_{i}}J=0$$

Provided that the solution is approximated by curves close to exponential ones, the calculation of the function $J=J\left(a_{1},\ldots ,a_{n}\right)$ corresponds to the use of asymptotic methods for calculating integrals containing a large parameter [44]. In this case, such a parameter is the ratio of the characteristic size of the system under consideration to the Debye length.

Thus, considering the accuracy of describing systems of this type using linearized equations in the long term also allows the use of asymptotic methods to describe systems that are promising in terms of the development of organic electronics [45], including electronics based on polyelectrolyte hydrogels [46]. The analysis of such systems is of interest, as noted above, from the point of view of creating neuromorphic materials, from which it is then possible to move on to sociomorphic ones [47,48,49,50].

The implementation of both neuromorphic and sociomorphic materials—at least at the level of practical application achieved by modern semiconductor technology—remains a matter for the future. At the present stage of research, it appears reasonable to consider, among other systems, simpler ones, in particular desalination devices based on the flow of a low-molecular-mass salt solution through a cross-linked polyelectrolyte network under the action of ordinary gravity [28]. In the cited work, it was shown that when a low-molecular-mass salt solution passes through a cross-linked polymer network, a potential difference arises at its cross-sections. The mechanism of this phenomenon is as follows. In the bulk of the cross-linked polyelectrolyte network immersed in a low-molecular-mass salt solution, ions of both signs are present; however, the concentration of one type is significantly higher, since ions of one sign are formed due to the dissociation of the functional groups of the network. For the condition of electrical neutrality of the sample to be satisfied, an electric field must arise that, to maintain balance, retards ions of one sign and accelerates ions of the opposite sign.

Phenomena of this kind can, of course, be investigated within the framework of consistent models; however, from the standpoint of qualitative interpretation, it is preferable to construct the primary model within the framework of the linearization procedure. Such a primary model may subsequently be used for the correct formulation of a refined model, for example, one constructed based on variational principles in accordance with the considerations presented above.

## 4. Conclusions

In the current study, we have demonstrated that the above traditional restriction for the linearized Poisson–Boltzmann equation, i.e., the condition of low electrostatic potentials, does not fully represent a necessary condition for the description of polyelectrolytes in the form of cross-linked networks in terms of linear equations. By establishing a linear equation directly from the electrostatic relations and the ion transport equations, we have shown that a linear mathematical description can formally be set up even without using the Boltzmann distribution in the form of a Taylor expansion.

The proposed equation keeps the Debye length as the dominant parameter and does not require the system to be close to thermodynamic equilibrium, as the ordinary linearized Poisson–Boltzmann equation does when an electric current exists in the system. This significantly expands the range of linear models’ applications in the physical chemistry of polyelectrolytes and similar systems.

A comparison between the exact solutions for the nonlinear equations and the approximate solutions using the linear approach was performed for a polyelectrolyte hydrogel–electrolyte interface. It has been found based on the potential difference and the electric field calculations that the difference between the two solutions remains acceptable for a wide concentration range, and significant variations occur only in low ionic strength solutions, where high potential differences are created, representing the applicability criterion as for the classic approach. The success of the model leads us to conclude that the use of linear differential equations in the theoretical modelling of polyelectrolyte hydrogels is well-justified in practical situations like organic electronic devices and in the design of neuromorphic devices. In a broader context, the present work has established a theoretical foundation for applying the logic of linear models even in non-equilibrium situations in polyelectrolyte systems.

## Data Availability

The original contributions presented in this study are included in the article. Further inquiries can be directed to the corresponding authors.

## Supplementary Materials

The following supporting information can be downloaded at: https://www.mdpi.com/article/10.3390/polym18050635/s1.
