# Supplementary material for: On the Accuracy of Describing Polyelectrolyte Systems Based on Cross-Linked Networks in Terms of Linear Differential Equations
Source: Polymers (Basel). 2026 Mar 4;18(5):635. doi: 10.3390/polym18050635 (PMC12986687; doi:10.3390/polym18050635)
Supplement: Supplementary file 1 [file polymers-18-00635-s001.zip › polymers-4174215-supplementary.pdf]

Supporting Materials S1

$$\frac{d}{dx}\left(\frac{1}{a}\ln\frac{a\sqrt{x^2+a^2}-a^2}{x}\right)=\frac{1}{a}\frac{x}{a\sqrt{x^2+a^2}-a^2}\frac{d}{dx}\left(\frac{a\sqrt{x^2+a^2}-a^2}{x}\right) \tag{S1}$$

$$\frac{d}{dx}\left(\frac{a\sqrt{x^2+a^2}-a^2}{x}\right)=-\frac{a\sqrt{x^2+a^2}-a^2}{x^2}+\frac{1}{x}\frac{ax}{\sqrt{x^2+a^2}} \tag{S2}$$

$$\frac{d}{dx}\left(\frac{a\sqrt{x^2+a^2}-a^2}{x}\right)=\frac{a^2\sqrt{x^2+a^2}-a(x^2+a^2)+ax^2}{x^2\sqrt{x^2+a^2}} \tag{S3}$$

$$\frac{d}{dx}\left(\frac{a\sqrt{x^2+a^2}-a^2}{x}\right)=a\frac{a\sqrt{x^2+a^2}-a^2}{x^2\sqrt{x^2+a^2}} \tag{S4}$$

$$\frac{d}{dx}\left(\frac{1}{a}\ln\frac{a\sqrt{x^2+a^2}-a^2}{x}\right)=\frac{1}{a}\frac{x}{a\sqrt{x^2+a^2}-a^2}a\frac{a\sqrt{x^2+a^2}-a^2}{x^2\sqrt{x^2+a^2}} \tag{S5}$$

$$\frac{d}{dx}\left(\frac{1}{a}\ln\frac{a\sqrt{x^2+a^2}-a^2}{x}\right)=\frac{1}{x\sqrt{x^2+a^2}} \tag{S6}$$

Supporting Materials S2

$$\ln\frac{2\sqrt{f^2+4}-4}{f}=-X+X_0 \tag{S7}$$

$$2\sqrt{f^2+4}-4=f\exp(-X+X_0) \tag{S8}$$

$$(4+f\exp(-X+X_0))^2=4f^2+16 \tag{S9}$$

$$f\exp 2(-X+X_0)-4f=-8\exp(-X+X_0) \tag{S10}$$

$$f=\frac{8\exp(-X+X_0)}{4-\exp 2(-X+X_0)} \tag{S11}$$
